# Co-Occurrence of 5-Hydroxymethylfurfural and Patulin in Reconstituted Pomegranate Juice: Analytical Determination and Risk Assessment

**DOI:** 10.3390/molecules31081309

**Published:** 2026-04-17

**Authors:** Cagla Kayisoglu

**Affiliations:** Scientific Technical Application and Research Center, Hitit University, Corum 19030, Türkiye; caglakayisoglu@hitit.edu.tr; Tel.: +90-364-2192850; Fax: +90-364-2192855

**Keywords:** pomegranate juice, food safety, mycotoxins, quality indicators, dietary exposure, hazard quotient, analytical method

## Abstract

5-Hydroxymethylfurfural (5-HMF) and the mycotoxin patulin (PAT) serve as crucial chemical markers for evaluating the quality and safety of fruit-derived beverages, particularly pomegranate juice. This study aimed to quantify the occurrence of 5-HMF and PAT in commercial reconstituted pomegranate juices and assess the associated dietary exposure risks. A total of 154 commercial samples, collected from a Turkish processing facility during the 2024–2025 production seasons, were analysed using high-performance liquid chromatography with diode-array detection. 5-HMF was detected in 152 samples (98.7%) at concentrations ranging from 1.03 to 10.79 mg/kg, with only two samples (1.3%) exceeding the critical threshold of 10 mg/kg. PAT was found in 57 samples (37.0%), with concentrations between 3.61 and 50.69 µg/kg, and only one sample (0.6%) exceeded the European Union maximum level established for fruit juices. Estimated mean daily intakes for adults and children ranged from 0.374 to 2.362 and 1.139 to 8.546 µg/kg bw/day for 5-HMF, and from 0.001 to 0.006 and 0.002 to 0.021 µg/kg bw/day for PAT, respectively. Risk characterisation based on hazard quotient values indicated that PAT exposure did not pose a significant health risk for either population group, highlighting the overall safety of the analysed products.

## 1. Introduction

Pomegranate (*Punica granatum* L.), a perennial small fruit tree belonging to the Punicaceae family, has long been recognized as a valuable species for both nutritional and medicinal purposes. It is widely cultivated across diverse geographic regions, particularly in tropical and subtropical climates. While China is currently the leading producer of pomegranates, other major contributors include India, Turkey, and the United States [1]. The fruit is rich in various bioactive compounds, including organic acids, sugars, vitamins, polysaccharides, polyphenols, and essential minerals. Due to this diverse nutrient profile, pomegranates are not only consumed fresh but are also widely processed into a wide range of food products such as juice, concentrate, canned beverages, jelly, jam, sauce, and syrup [2].

Among pomegranate-derived products, juice and juice concentrate are considered the primary sources through which its bioactive constituents are consumed. One of the key quality indicators for pomegranate juice and its concentrates is 5-hydroxymethylfurfural (5-HMF), a process contaminant generated during thermal treatment. This furan derivative is formed either as an intermediate product of the Maillard reaction, involving interactions between hexoses and amino compounds, or via direct dehydration of sugars during caramelization. The formation of 5-HMF is strongly influenced by factors such as sugar type and concentration, the presence of acidic conditions, temperature, heating duration, pH, water activity, and antioxidant levels [3]. Accumulation of 5-HMF during processing can lead to degradation of anthocyanin pigments and diminished antioxidant capacity, thereby reducing the nutritional value and contributing to browning in pomegranate juice. This undesirable color change adversely affects the visual appeal of the product and significantly lowers consumer acceptance [4]. In addition to its impact on quality, 5-HMF has also been shown to exert toxicity in animal experiments and may have potentially adverse effects in humans. Furthermore, 5-HMF is known to be toxic to bees, with HMF-containing bee feed having been associated with reduced lifespan and increased mortality in apicultural settings [5,6].

Although no maximum level (ML) for 5-HMF content in fruit juices or juice concentrates has been established by either the European Union (EU) or the Turkish Food Codex, several industry-specific guidelines have been proposed to ensure product quality and safety. In this regard, the European Fruit Juice Association (AIJN) has stipulated a maximum 5-HMF concentration of 20 mg/kg for fruit juice concentrate [7], aligning with China’s national standard GB/T 18963-2012 [3]. Moreover, the International Federation of Fruit Juice Processors (IFFJP) has recommended that the 5-HMF content in fruit juices should remain within the range of 5–10 mg/kg, and should not exceed 25 mg/kg in juice concentrates [8].

Another important quality indicator in fruit juices and their concentrates is patulin (PAT), a mycotoxin produced primarily by fungal species of *Penicillium*, *Aspergillus*, and *Byssochlamys*. Among these fungi, *Penicillium expansum* has been identified as the principal PAT-producing species, commonly associated with blue mold decay found in rotten fruits during storage. However, *P. expansum* is also capable of developing on the surface of intact fruits and poses a contamination risk in orchards and postharvest environments, particularly in damaged fruits that have been compromised by other microorganisms [9].

PAT contamination can occur in various fruits, such as apples, oranges, pomegranates, peaches, and apricots, as well as in their derived products during growth, harvest, or storage [10]. The use of mold-infected fruits in the production of juice and concentrates has been recognized as a main contributor to PAT contamination in processed products. Although certain conditions, such as high temperatures, prolonged storage, and pasteurization, have been reported to reduce PAT levels to some extent, complete degradation of the toxin during thermal processing is rarely achieved [11].

From a toxicological standpoint, PAT has been associated with a broad spectrum of adverse health effects, primarily affecting the liver, spleen, and kidneys upon repeated oral exposure in experimental animals [9]. High-level exposure has been linked to gastrointestinal inflammation, pulmonary congestion, oedema, and neurological manifestations such as agitation and convulsions [11]. Furthermore, PAT has been shown to disrupt protein synthesis, inhibit DNA and RNA polymerases, and form covalent adducts with thiol-containing biomolecules, thereby contributing to oxidative stress, neurotoxicity, and immunotoxicity Despite exhibiting embryotoxic and immunosuppressive properties, no evidence of reproductive toxicity or teratogenicity has been observed in rodents at oral doses of up to 1.5 mg/kg bw/day [12]. While its genotoxic potential remains controversial, PAT has been considered non-carcinogenic in humans and is currently classified by the International Agency for Research on Cancer (IARC) in Group 3, “not classifiable as to its carcinogenicity to humans” [13]. Based on a no-observed-effect level (NOEL) of 43 µg/kg bw/day identified in long-term rat studies and applying a safety factor of 100, a provisional maximum tolerable daily intake (PMTDI) of 0.4 µg/kg bw/day has been established by the Joint FAO/WHO Expert Committee on Food Additives (JECFA) [12].

Although several studies have investigated the formation and levels of 5-HMF during the production of pomegranate juice [14,15,16], data on PAT contamination in pomegranate juice remain extremely limited. The aim of this study was therefore to address this knowledge gap by assessing the concentrations of two critical indicators, 5-HMF, a marker of product quality, and PAT, a mycotoxin of toxicological concern, in pomegranate juice concentrates produced in Turkey. A total of 154 samples were analyzed using high-performance liquid chromatography with diode-array detection (HPLC-PDA). In addition to determining the occurrence levels of these compounds, the study also involved a chronic dietary exposure and risk assessment for both adults and children, in accordance with established toxicological reference values. The influence of factors such as raw material quality, thermal processing techniques, and storage conditions was also discussed, providing valuable evidence on the contamination risks inherent in pomegranate juice production and contributing to a broader understanding of food safety challenges in fruit-based products.

## 2. Results and Discussion

### 2.1. Validation Data

A summary of the method validation parameters, including linearity, LOQs, recoveries, and RSDs, is presented in Table 1. Calibration curves exhibited excellent linearity for both target analytes, with correlation coefficients (*R*^2^) exceeding 0.99 across the tested concentration ranges. The LOQs, calculated as ten times the standard deviation of replicate analyses of spiked blank matrix samples, were determined to be 1 mg/kg for 5-HMF and 3.5 μg/kg for PAT. The LOQ value of 1 mg/kg was well below the commonly applied quality criterion of 10 mg/kg for 5-HMF. For PAT, the LOQ of 3.5 μg/kg complied with the requirements set by Commission Implementing Regulation (EU) 2023/2782, which recommends an LOQ not exceeding 0.5 times the ML (≤25 μg kg^−1^) and preferably below 0.2 times the ML (≤10 μg/kg) [17], thereby confirming the method’s high sensitivity.

Method accuracy was verified through recovery experiments at two fortification levels for each compound. The mean recoveries ranged from 97.50% to 101.32% for 5-HMF and from 82.54% to 89.71% for PAT, demonstrating satisfactory trueness across the tested levels. Precision, expressed as intra-day repeatability (*n* = 6, RSDs), ranged between 5.60% and 6.45% for 5-HMF and between 7.62% and 9.27% for PAT. The recovery values and RSDs obtained for PAT fell within the specific performance criteria defined for confirmatory methods under Commission Implementing Regulation (EU) 2023/2782, which stipulates an acceptable recovery range of 70–120% and a precision threshold of no more than 20% [17].

### 2.2. Occurrence of 5-HMF in Reconstituted Pomegranate Juice

The concentrations of 5-HMF detected in reconstituted pomegranate juice samples are presented in Table 2. In the 2024 production period, 5-HMF was quantified in all 105 reconstituted juice samples, with concentrations ranging from 1.16 to 10.79 mg/kg and an average level of 3.55 mg/kg. Among these, only two samples were found to exceed the 10 mg kg^−1^ threshold. In contrast, among the 49 samples collected in 2025, 5-HMF was detected in 47 samples (95.9%) at concentrations between 1.03 and 4.36 mg/kg, with a mean value of 2.25 mg/kg.

**Figure 1:**
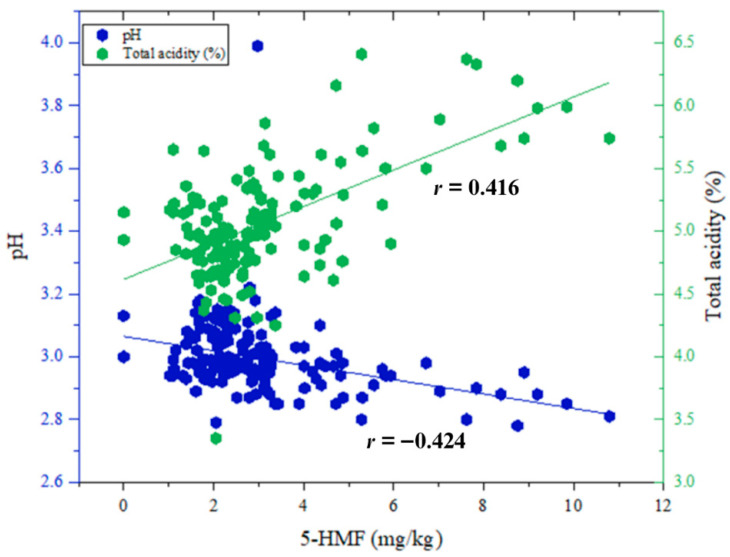
Correlation between 5-HMF concentrations and pH and total acidity in pomegranate juice samples.

The pH values of the pomegranate juice samples ranged from 2.79 to 3.99, reflecting an acidic environment that is known to facilitate the formation of 5-HMF. A statistically significant moderate negative correlation was observed between 5-HMF concentration and pH (Spearman’s *r* = −0.424, *p* < 0.01), indicating that lower pH levels may be associated with increased 5-HMF formation (Figure 1). This finding aligns with previous research by Córdova et al., who reported increased 5-HMF concentrations at reduced pH during the storage of fruit-based products such as apple juice [18]. Although the fruit matrix differs, the underlying chemical mechanism whereby acidic conditions accelerate the acid-catalyzed dehydration of hexoses remains consistent across different fruit systems. The acidic conditions, particularly at pH values below 4.0, are known to promote the acid-catalyzed dehydration of hexoses such as fructose and glucose, which are abundant in pomegranate juice. Under these conditions, 5-HMF is formed via pathways involving the loss of water molecules from sugar precursors. In addition, low pH may enhance the reactivity of Maillard reaction intermediates, further contributing to 5-HMF accumulation. Furthermore, a statistically significant moderate positive correlation was identified between 5-HMF concentrations and total acidity (*r* = 0.416, *p* < 0.01), suggesting that higher acid content may also play a contributory role in the enhancement of 5-HMF levels (Figure 1). This relationship underscores the multifactorial influence of acidity, encompassing both free hydrogen ion concentration and the overall acidic composition of the juice matrix on 5-HMF formation. The relatively low 5-HMF levels detected in our samples, compared to the broader range (0.12 to 67.22 mg/kg) reported by Vatansever et al. [19], might be explained by more controlled thermal processing conditions or differences in the initial sugar profiles of the concentrates used. Notably, the relatively wide range of pH and total acidity values observed among the samples may partly account for the variability in 5-HMF concentrations, especially in the 2024 production period, during which some samples exceeded the 10 mg/kg threshold.

The 5-HMF levels observed in the present study appear lower than those previously reported in commercial pomegranate juice samples. In a study conducted in Turkey by Vatansever et al., 5-HMF levels in commercially available pomegranate juice samples were found to range from 0.12 to 67.22 mg/kg [19]. The relatively low 5-HMF levels detected in our samples, compared to the broader range (0.12 to 67.22 mg/kg) reported by Vatansever et al. [19], might be explained by more controlled thermal processing conditions or differences in the initial sugar profiles of the concentrates used. Instead of a broad comparison with other fruit matrices, the 5-HMF levels in this study should be interpreted through the specific commercial production steps shown in Figure 1. The ‘Heat treatment (85–95 °C)’ and ‘Pre-concentration’ stages are the primary thermal drivers for 5-HMF formation. The relatively low values observed (mean of 3.55 mg/kg in 2024 and 2.25 mg/kg in 2025) suggest that the thermal history during the concentration of these specific pomegranate juices was well-regulated, minimizing the acid-catalyzed dehydration of hexoses despite the low pH environment (2.79–3.99) and the final reconstitution to 15 °Brix. Similarly, in another study, 5-HMF concentrations in four pomegranate juice samples varied between 5.5 and 27.4 mg/kg, with two samples containing less than 10 mg/kg and the others exceeding this threshold. In comparison, 5-HMF concentrations detected in apple, orange, and grape juice samples ranged between 1.62–7.49 mg/kg, 0.35–0.58 mg/kg, and 0.34–24.38 mg/kg, respectively, indicating that pomegranate juice generally contained higher 5-HMF levels than other fruit juices [20]. This difference may be attributed to the higher content of reducing sugars and amino acids in pomegranate juice, as well as the thermal processing conditions commonly applied during its production, which are known to promote 5-HMF formation via the Maillard reaction and sugar dehydration pathways.

The influence of thermal processing methods on 5-HMF formation in pomegranate juice has been widely reported in the literature and may help to explain the relatively low levels observed in the present study. For example, Sabanci et al. demonstrated that short-duration, high-voltage ohmic heating resulted in significantly lower 5-HMF levels compared to longer, low-voltage treatments [21]. In addition, ohmic heating has been reported to generate less 5-HMF than conventional vacuum evaporation, with levels ranging from 2.70 to 5.40 mg/kg. These findings suggest that milder or more controlled processing conditions may contribute to limiting 5-HMF formation.

Similarly, extreme thermal treatments have been associated with substantial increases in 5-HMF concentration. Ersus et al. reported that prolonged heating of pomegranate juice at 200 °C led to an approximately 100-fold increase in 5-HMF levels [15]. Although processing parameters were not directly assessed in the present study, the relatively low concentrations detected may reflect the use of controlled industrial processing conditions, as supported by previous findings.

The correlation between temperature and 5-HMF formation has been further demonstrated by Fischer et al., who reported a substantial increase in 5-HMF levels with rising heating temperatures [14]. Specifically, when pomegranate juice was heated at 60 °C, the 5-HMF concentration was measured at 4.6 mg/kg, while heating at 70 °C, 80 °C, and 90 °C led to increases to 9.9 mg/kg, 11.4 mg/kg, and 12.5 mg/kg, respectively, indicating an increase in 5-HMF formation of up to 170% with rising temperatures.

The method of concentration also plays a critical role in 5-HMF formation. In a comparative study, Trishitman et al. observed that thermal evaporation led to a significant increase in 5-HMF levels during accelerated storage, from 16.76 mg/kg to 133.68 mg/kg [16]. In contrast, only a moderate increase in 5-HMF content, from 3.64 mg/kg to 15.79 mg/kg, was observed following concentration by forward osmosis, a membrane-based technique. These findings highlight the potential of non-thermal membrane technologies to substantially mitigate 5-HMF formation during concentration processes.

The effect of phenolic compounds on 5-HMF formation has been demonstrated in a study by Türkyılmaz et al., in which the addition of phenolic acids such as ferulic, gallic, and caffeic acids to pomegranate juice significantly reduced 5-HMF levels by up to 60% [4]. While phenolic content was not specifically measured in this study, the inherently high phenolic profile of pomegranate juice may have acted as a natural inhibitor, potentially contributing to the generally low HMF levels observed in our 2024 and 2025 samples. These results suggest that phenolic compounds not only contribute to the antioxidant capacity of the juice but also serve as inhibitors of undesirable Maillard reaction products like 5-HMF. Moreover, ferulic acid was reported to limit anthocyanin degradation induced by 5-HMF, thereby contributing to improved color stability during processing.

### 2.3. Occurrence of PAT in Reconstituted Pomegranate Juice

The occurrence and levels of PAT in reconstituted pomegranate juice samples (~15.0 °Brix) are summarized in Table 3. PAT was detected in 57 out of 154 analyzed samples (37.0%) at concentrations ranging from 3.61 to 50.69 µg/kg (mean = 14.48 µg/kg), while the remaining 97 samples (73.0%) were free of detectable PAT. Only one sample (0.6%) exceeded the EU ML of 50 µg kg^−1^ [22]. Among the 105 samples collected in 2024, PAT was found in 18 samples (17.1%) at levels ranging from 3.61 to 24.09 µg/kg, with a mean level of 9.90 µg/kg. In contrast, among the 49 samples collected in 2025, PAT was detected in 39 samples (79.6%) within the range of 3.71–50.69 µg/kg, with a mean concentration of 16.59 µg/kg.

A significant increase in PAT incidence was observed in samples produced in 2025 compared to those from 2024, with detection frequencies rising from 17.1% to 79.6%, respectively. Although all pomegranate juice samples originated from the same harvest season, this marked difference may be attributed to differences in the postharvest storage period of the raw fruits prior to juice production. Specifically, the 2024 samples were produced between 4 October and 30 December 2024, while the 2025 samples were manufactured between 4 January and 11 March 2025. Given that pomegranate harvest in Turkey typically ends by late November or early December, it is likely that fruits used in the production of the 2025 samples had been stored for extended periods under postharvest conditions. This prolonged storage may have created favorable conditions for fungal proliferation and PAT biosynthesis, especially in the presence of mechanical damage or latent infections. It is possible that environmental parameters during storage, such as high humidity or fluctuating temperatures, could have promoted the growth of PAT-producing fungi, particularly Penicillium expansum, which are known to thrive under such conditions. Although these parameters were not directly monitored in this study, the extended storage duration likely increased the susceptibility of the fruit to fungal proliferation. Moreover, extended storage has been linked to physiological deterioration in fruit tissue, increasing susceptibility to microbial invasion. The substantial difference in contamination rates between the two production periods highlights the critical influence of storage duration and conditions on PAT occurrence in fruit-based products.

**Figure 2:**
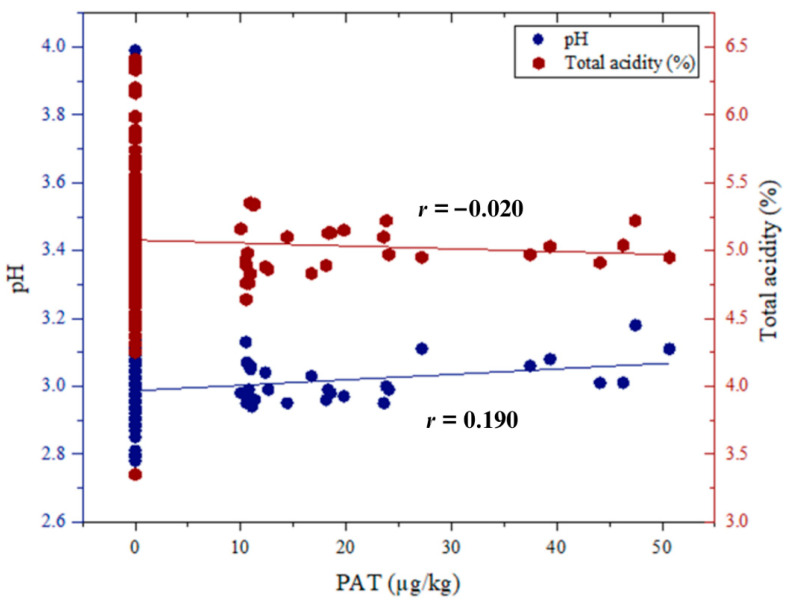
Correlation between PAT concentrations and pH and total acidity in pomegranate juice samples.

Beyond storage-related factors, the role of intrinsic juice parameters in PAT accumulation was also investigated. In particular, a very weak positive correlation was identified between PAT concentrations and pH values (r = 0.190, *p* < 0.01) (Figure 2). Although this correlation is statistically significant, the extremely low correlation coefficient clearly indicates that its biological and technological significance is highly limited. Given that 63% of the samples remained below the LOQ, this statistical association primarily reflects a marginal trend within the censored dataset. While previous observations in apple-based matrices have linked PAT accumulation to pH values approaching 4.0 [23,24], care must be taken not to overinterpret this relationship in the present study. The current data suggest that within the highly acidic environment of pomegranate juice (pH 2.79–3.99), pH exerts only a negligible, non-primary influence on PAT levels compared to overriding factors such as prolonged storage.

In contrast, the correlation between PAT concentrations and total acidity was found to be negative (Figure 2) and negligible (*r* = −0.020), with no statistical significance. This suggests that total acidity may exert only a limited or indirect influence on PAT dynamics within this matrix. The absence of a significant relationship, despite the weak association observed with pH, may reflect the fact that total acidity represents the total acid content rather than the active hydrogen ion concentration. As such, it may not directly impact fungal metabolic activity or PAT biosynthesis in the same manner as pH, especially in matrices characterized by high acidity, such as pomegranate juice. While a statistically significant correlation was observed (r = 0.190), its biological relevance appears limited given the weak nature of the association. This suggests that pH may only have a marginal, non-primary influence on PAT levels in this specific matrix.

Although the production and consumption of pomegranate juice have increased markedly in recent years, the potential risk associated with mycotoxin contamination in this product has remained underexplored, representing a significant gap in terms of consumer protection. Pomegranate is often considered inherently resistant to fungal spoilage due to its low pH, high phenolic content, and robust peel structure. However, the detection of PAT in a substantial proportion of samples indicates that these natural defenses may be compromised under certain conditions. PAT contamination is most commonly associated with physically damaged, decayed, or improperly stored fruits. The growth of *P. expansum*, the primary PAT producer, is favored by conditions such as temperatures ranging from 0 to 25 °C, relative humidity around 90%, and water activity levels approaching 0.99 [9]. These conditions can develop when fruits are stored for prolonged periods without adequate environmental controls.

Potential interaction between PAT and 5-HMF levels was also assessed to gain further insight into the influence of processing conditions. Although a negative correlation was observed between PAT and 5-HMF concentrations (r = −0.081), this relationship was both very weak and statistically non-significant (*p* > 0.05). The absence of a significant correlation suggests that variations in 5-HMF levels may not consistently influence PAT dynamics in pomegranate juice. While it has been hypothesized that high 5-HMF concentrations may reflect processing environments that suppress fungal viability and toxin biosynthesis, the weak and non-significant nature of the observed relationship implies that PAT and 5-HMF are not necessarily governed by shared physicochemical parameters in this matrix.

While most studies in Turkey and globally have focused on PAT contamination in apple juice due to its high susceptibility [25,26,27], our findings demonstrate that pomegranate juice also requires close monitoring despite its perceived resistance to fungal growth. In apple-based matrices, PAT synthesis is known to be optimized around pH 4.0 and influenced by storage conditions [9,24], a trend that aligns with the weak but significant pH-related accumulation observed in our pomegranate samples. International studies, particularly from Iran, have reported mean PAT levels in pomegranate juice ranging from 4.2 to 8.3 µg/kg [28,29]. Our results, with an average of 14.48 µg/kg, indicate a higher occurrence level, suggesting that factors such as raw material quality, storage duration, and processing practices remain critical even for fruits with high acidity and phenolic content. These comparisons emphasize that pomegranate juice is not exempt from mycotoxin risks and necessitates established control mechanisms similar to those used for more susceptible fruits. These findings suggest that raw material quality, storage duration, and processing practices critically influence PAT levels, even in fruits with strong inherent resistance to microbial contamination.

### 2.4. Risk Assessment

The estimated exposure levels to 5-HMF and PAT through pomegranate juice consumption under two different scenarios are summarized in Table 4. To account for uncertainty in samples with concentrations below the limit of quantification (LOQ), a substitution method was applied according to EFSA recommendations. Specifically, three scenarios were considered: the lower bound (LB) where non-detects were replaced with zero, the middle bound (MB) where they were replaced with LOQ/2, and the upper bound (UB) where they were replaced with the value of the LOQ.

It should be noted that, due to the absence of specific consumption data for pomegranate juice, fruit juice intake data were used as a surrogate for exposure calculations. This substitution may result in a degree of overestimation of actual 5-HMF and PAT intake, and thus the findings should be interpreted with appropriate caution.

The mean chronic daily intake values of 5-HMF for adults were estimated to range from 2.352 to 2.362 µg/kg bw/day (LB to UB) under Scenario 1, and from 0.374 to 0.376 µg/kg bw/day under Scenario 2. The corresponding 95th percentile (P95) exposure levels were calculated as 5.828 µg/kg bw/day in Scenario 1 and 0.927 µg/kg bw/day in Scenario 2. In comparison, children exhibited higher exposure levels across both scenarios. Mean intake values ranged from 8.510 to 8.546 µg/kg bw/day in Scenario 1 and from 1.139 to 1.144 µg/kg bw/day in Scenario 2, while P95 exposure levels were estimated at 21.08 µg/kg bw/day and 2.821 µg/kg bw/day, respectively. Notably, for both adults and children, exposure levels under Scenario 1 were approximately sixfold higher than those estimated under Scenario 2, reflecting the influence of varying consumption assumptions between the two dietary scenarios.

In the absence of an established reference dose (*RfD*) or health-based guidance value for 5-HMF, risk characterization was approached through comparison with theoretical benchmarks available in the scientific literature. The Scientific Panel on Food Additives, Flavourings, Processing Aids and Materials in Contact with Food developed the theoretical maximum added daily intake (mTAMDI) framework, which provides per capita daily intake estimates in the absence of accurate toxicological thresholds. The mTAMDI value for 5-HMF is reported as 1.6 mg/person/day, with a threshold of concern set at 540 µg/person/day The *NEDI* of 5-HMF calculated in this study did not exceed both the mTAMDI and threshold of concern values. Furthermore, the EFSA concluded, based on a benchmark dose lower confidence limit (BMDL) of 14.4 mg/kg bw/day derived from a 13-week rodent study, that 5-HMF does not pose a safety concern as a flavoring substance at its current levels of use in foods [5]. In addition to these theoretical benchmarks, the detected 5-HMF levels were evaluated against the quality criteria established by the AIJN (European Fruit Juice Association) Code of Practice, which is the internationally recognized reference for fruit juice quality standards followed by the Turkish fruit juice industry and regulatory frameworks. According to the AIJN Reference Guideline for Pomegranate Juice, the maximum guidance limit for 5-HMF is set at 20 mg/L [4]. In the present study, the average 5-HMF level was found to be 3.55 mg/kg (ranging from 1.16 to 10.79 mg/kg in 2024 and 1.03 to 4.36 mg/kg in 2025), which is substantially lower than this industrial quality threshold. This high level of compliance with both national and international standards further confirms that the 5-HMF concentrations in commercially available pomegranate juices in Turkey do not pose a significant health risk or quality concern, reflecting well-controlled thermal processing conditions.

The mean chronic daily intake of PAT for adults ranged from 0.004 to 0.006 µg/kg bw/day (LB–UB) under Scenario 1, and was 0.001 µg/kg bw/day (LB–UB) under Scenario 2, with corresponding P95 values of 0.018 and 0.003 µg/kg bw/day, respectively. In children, exposure levels were considerably higher: mean intakes ranged from 0.015 to 0.021 µg/kg bw/day under Scenario 1, and from 0.002 to 0.003 µg/kg bw/day under Scenario 2, with P95 values of 0.066 and 0.009 µg/kg bw/day, respectively. These values fell well below the PMTDI for PAT established by the JECFA, which is set at 0.4 µg/kg bw/day [12]. Under the UB scenario, HQ values for adults were calculated as 0.014 (Scenario 1) and 0.002 (Scenario 2) for the mean, and 0.045 (Scenario 1) and 0.007 (Scenario 2) for the P95 exposure. For children, HQs were 0.051 (Scenario 1) and 0.007 (Scenario 2) for the mean, and 0.164 (Scenario 1) and 0.022 (Scenario 2) for the P95. All HQ values were well below the benchmark value of 1, indicating negligible health risk under both exposure scenarios.

Although data on PAT exposure from pomegranate juice consumption remain limited in the scientific literature, a study conducted in Iran evaluated PAT intake from various fruit juices and reported that pomegranate juice contributed less to PAT exposure than apple, orange, peach nectar, and pineapple juices, but more than sour cherry, mango, and grape juices [28]. In that study, the estimated dietary intake of PAT from pomegranate juice was 0.0012 µg/kg bw/day, accounting for 0.38% of the PMTDI. This estimate is approximately 4.8-fold lower than the mean UB intake calculated under Scenario 1 in the present study. It should be noted, however, that in the absence of specific consumption data for pomegranate juice, the present study relied on generalized fruit juice consumption scenarios. Therefore, the observed differences may stem not only from regional consumption patterns, analytical methods, or processing variations, but also from differences in exposure assessment approaches.

In the broader context of PAT exposure, the majority of published studies have concentrated on apple juice, due to its well-documented susceptibility to patulin contamination. Intake levels of PAT from apple juice have shown considerable geographic variation, ranging from as low as 0.0003 µg/kg bw/day in France [30] to 0.0164 µg/kg bw/day in Iran [29]. In a previous study conducted by the author, PAT exposure among adults from apple juice consumption in Turkey was estimated to range between 0.000083 and 0.000091 µg/kg bw/day [25]. These values were found to be approximately 50–63 times lower than the highest level reported in the present study under Scenario 1, and 8–10 times lower under Scenario 2. However, it is crucial to emphasize that these cross-study comparisons must be interpreted with strict caution. Because the present exposure estimates rely on general fruit juice consumption data rather than pomegranate-specific intake rates, direct comparisons likely overestimate the relative risk of pomegranate juice compared to matrices like apple juice, which have specific and established, often modest, consumption metrics [31]. Therefore, the observed differences primarily highlight the methodological limitations of using surrogate consumption data rather than confirming an elevated risk profile for pomegranate juice. These findings emphasize the substantial geographic variation in PAT exposure from fruit juices, which appears to be closely linked not only to the quality of raw fruits used in juice production but also to local consumption patterns and dietary habits.

## 3. Materials and Methods

### 3.1. Reagents, Chemicals, and Standards

HPLC-grade methanol and acetonitrile were obtained from Sigma-Aldrich (St. Louis, MO, USA). Ethyl acetate was purchased from Supelco (Darmstadt, Germany). Anhydrous sodium carbonate (Na_2_CO_3_), anhydrous sodium sulfate (Na_2_SO_4_), and perchloric acid (HClO_4_) were supplied by Merck (Darmstadt, Germany). Ultrapure water was produced using a Milli-Q purification system (Millipore, Molsheim, France).

Analytical standards of 5-HMF and PAT, each with a purity of ≥98%, were procured from Sigma-Aldrich (St. Louis, MO, USA). To prepare the 1000 μg/mL stock solution of 5-HMF, the solid standard was dissolved in water and stored at −18 °C until use. Working standard solutions were prepared by appropriate dilution of the stock solution with water. For PAT, a 100 μg/mL stock solution was prepared by dissolving the standard in ethyl acetate and stored at −18 °C. An intermediate solution at a concentration of 10 μg/mL was obtained by evaporating an aliquot of the stock solution under a gentle stream of nitrogen, followed by reconstitution in water acidified to pH 4.0 with sulfuric acid (H_2_SO_4_). This intermediate solution was subsequently diluted with water adjusted to pH 4.0 to prepare the calibration standards.

### 3.2. Sampling of Pomegranate Juice Samples

**Figure 3:**
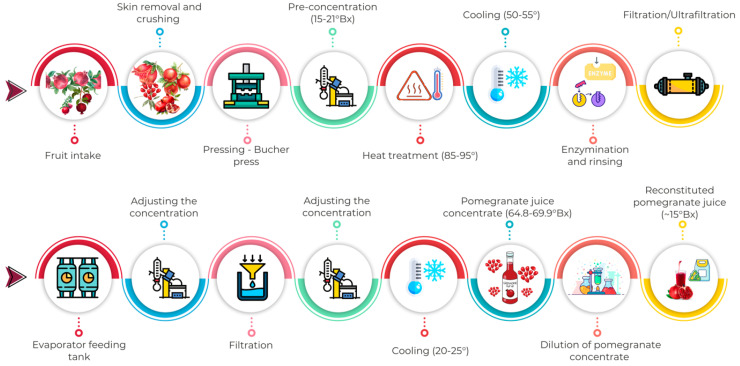
Commercial production steps of reconstituted pomegranate juice.

A total of 154 reconstituted pomegranate juice samples (~15 °Brix) from concentrated fruit juice (64.8–69.9 °Brix) were collected from a fruit juice processing facility operating in Turkey. The samples were obtained from commercial production batches between 4 October and 30 December 2024 (*n* = 105), and between 4 January and 11 March 2025 (*n* = 49). The production flow chart of reconstituted pomegranate juice is presented in Figure 3. Each sample was collected in 1 L portions in its original packaging and transported to the laboratory. Upon arrival, the samples were stored at 4 ± 1 °C. All analyses were carried out within 24–48 h of sample collection.

### 3.3. Chemical Analysis

Soluble solids content (°Brix), pH, and total acidity were determined according to the official methods described by the Association of Official Analytical Chemists (AOAC) [32]. The soluble solids content was measured using a digital refractometer (Atago PAL3, Tokyo, Japan). The pH was determined with a calibrated pH meter (Adwa AD1020, Szeged, Hungary). Total acidity was measured by titration with 0.1 N NaOH to an endpoint of pH 8.2 and the results were expressed as % of citric acid equivalent.

### 3.4. Sample Preparation

#### 3.4.1. 5-HMF

The reconstituted pomegranate juice samples were filtered through a 0.45 µm membrane filter. The filtrate was then transferred into HPLC vials for analysis.

#### 3.4.2. PAT

**Figure 4:**
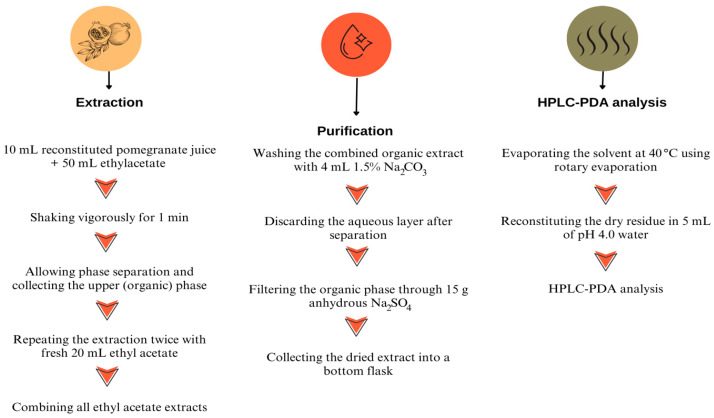
Extraction procedure for PAT analysis in pomegranate juice.

The extraction of PAT in reconstituted pomegranate juice was performed with minor modifications based on the AOAC Official Method 2000.02 [33]. The extraction procedure of PAT is illustrated in Figure 4. Briefly, 10 mL of reconstituted pomegranate juice (~15.0 °Brix) was extracted three times with 20 mL of ethyl acetate by shaking vigorously for 1 min using a vortex mixer. After phase separation, the combined ethyl acetate phases were subjected to an alkaline wash with 4 mL of sodium carbonate solution (1.5%) by shaking for 1 min. After allowing the phases to separate, the aqueous phase was discarded, and the organic phase was passed through a filter containing 15 g of anhydrous sodium sulfate and collected into a round-bottom flask. The filtrate was evaporated to dryness using a rotary evaporator at 40 °C until all ethyl acetate had been completely removed. The resulting dry residue was reconstituted in 5 mL of water adjusted to pH 4, yielding a final extract ready for HPLC injection.

### 3.5. HPLC Analysis

The qualitative and quantitative analyses of 5-HMF and PAT in reconstituted pomegranate juice samples were carried out using a Shimadzu LC-20A modular HPLC system (Tokyo, Japan) equipped with a Shimadzu SPD-M20A PDA detector. Reversed-phase separation was carried out on an InertSustain Inertsil ODS-3 column (150 × 4.6 mm, 5 µm; GL Sciences, Tokyo, Japan). All chromatographic runs were conducted under isocratic conditions.

For the determination of 5-HMF, a mobile phase consisting of 10% methanol, 90% water, and 0.01% acetic acid was employed. The flow rate was maintained at 0.8 mL/min, and a 20 μL aliquot of the sample was injected into the system. Detection was performed at 280 nm using the PDA detector.

For the analysis of PAT, a mobile phase composed of 5% acetonitrile, 95% water (adjusted to pH 4.0), and 0.095% perchloric acid was used. The flow rate was set at 1.0 mL/min, and a 50 μL sample volume was injected. Detection was carried out at 276 nm with the PDA detector.

### 3.6. Method Validation

The performance of the analytical method was evaluated in accordance with EU guidelines [17] and the detailed validation procedures for PAT described by Golge et al. [25]. Linearity was assessed by constructing calibration curves at seven concentration levels for 5-HMF (0.5–80 mg/kg) and six levels for PAT (2.5–250 μg/kg). The limits of quantification (LOQs), as well as accuracy and precision, were determined using spiked pomegranate juice samples. The LOQs were calculated as ten times the standard deviation of replicate analyses of the blank matrix spiked with 5-HMF (5 mg/kg) and PAT (5 μg/kg). Accuracy was evaluated through recovery studies at two fortification levels: 5 and 20 mg/kg for 5-HMF, and 10 and 50 μg/kg for PAT. Precision was expressed as repeatability and was assessed through six replicate (*n* = 6) intra-day analyses. Variability was determined by calculating the relative standard deviations (RSDs%) of the measured concentrations.

### 3.7. Estimated Daily Intake and Risk Assessment

The national estimated daily intake (*NEDI*) of 5-HMF and PAT for adults and children through pomegranate juice consumption was determined using Equation (1).

(1) $$NEDI=\;\frac{Concentration\;of\;residue\;\left(\mu g/kg\right)\;\times \;Pomegranate\;juice\;consumption\;(kg)}{Body\;weight\;(kg)}$$

In the absence of specific data on pomegranate juice consumption, two distinct exposure scenarios were considered to ensure a conservative and comprehensive intake assessment. In Scenario 1, available data on fruit juice consumption in EU populations were used. According to a comprehensive survey encompassing nine countries, the mean fruit juice intake among children ranged from 21 to 97 g/day (mean = 63 g/day). For adults, population mean intakes reported across 14 countries ranged from 24 to 115 g/day (mean = 53 g/day) [34]. In Scenario 2, the average per capita fruit and vegetable juice consumption was taken as 8.43 g/day, based on the G06 cluster diets defined by the GEMS/Food Consumption Cluster Diets database [31], maintained by the World Health Organization (WHO). For both scenarios, standard body weight (bw) values of 70 kg for adults and 23 kg for children aged 3–10 years were applied, in accordance with the recommendations of the EFSA [35].

To address the uncertainty associated with left-censored data, i.e., residue levels below the LOQ, a substitution method was applied in accordance with EFSA guidelines [36]. Specifically, three exposure scenarios were constructed: Lower Bound (LB, with non-detects assumed to be 0), Middle Bound (MB, with non-detects assumed to be LOQ/2), and Upper Bound (UB, with non-detects assumed to be LOQ).

In addition, to evaluate the potential health risks associated with dietary exposure to PAT, the hazard quotient (*HQ*) was calculated by dividing the *NEDI* of PAT by the provisional maximum tolerable daily intake (*PMTDI*) of 0.4 μg/kg bw/day established by the World Health Organization [12], as shown in the equation below:

(2) $$HQ=\;\frac{NEDI\;of\;PAT\;(\mu g/kg\;bw/day)}{PMTDI\;(0.4\;\mu g/kg\;bw/day)}$$

An *HQ* value exceeding 1 was interpreted as indicative of a potential health concern, while values equal to or below 1 were considered to represent an acceptable level of risk.

### 3.8. Statistical Analysis

Spearman’s rank correlation analysis was conducted to assess associations between 5-HMF, PAT, and chemical parameters, including pH and total acidity, in reconstituted pomegranate juice samples. For statistical evaluation, values below the LOQ were substituted with zero, following the LB approach. All statistical analyses were performed using SPSS^®^ Statistics version 27, with statistical significance considered at *p* < 0.05 and *p* < 0.01 levels.

## 4. Conclusions

This study provides novel insights into the occurrence and dietary exposure of 5-HMF and PAT in commercial pomegranate juice, addressing a critical gap in the literature. The findings confirm that dietary exposure to both compounds remains consistently below established toxicological thresholds, indicating a negligible health risk for adults and children. Specifically, the consistently low levels of 5-HMF reflect well-managed thermal processing conditions and demonstrate high compliance with international industry quality standards. However, the detection of PAT underscores the critical importance of raw material quality and postharvest storage. Effective risk management necessitates strict adherence to critical control points during production particularly the monitoring of storage duration and environmental conditions to prevent fungal proliferation even in inherently acidic fruits. Furthermore, the reliance on surrogate fruit juice intake data in this study highlights the necessity for pomegranate-specific consumption metrics to refine future risk assessments. These findings could provide useful data for regulatory agencies to consider within broader food safety monitoring frameworks. Although this study focuses on a single large-scale processing plant, providing a detailed case study of commercial production, future monitoring efforts across multiple enterprises with varying processing techniques will enhance the generalizability of these findings to the entire market. Ultimately, integrating temporally resolved consumption data and multi-contaminant analysis will ensure more robust consumer protection.

## Figures and Tables

**Table 1:** Validation parameters for 5-HMF and PAT in reconstituted pomegranate juice.

Analyte	LOQ (mg/kg)	Spike Level (mg/kg)	Recovery (%)	Intra-Day Repeatability, %RSD (*n* = 6)
5-HMF	1.0	5.0	101.32	5.60
		20.0	97.50	6.45
PAT	0.0035	0.010	82.54	7.62
		0.050	89.71	9.27

**Table 2:** Distribution and concentrations of 5-HMF in reconstituted pomegranate juice samples.

Food Products	Parameters	5-HMF
Pomegranate juice (*n* = 154)	Positive samples ^a^ *n* (%)	152 (98.7)
	Range (min–max, mg/kg)	1.03–10.79
	Mean of positive samples (mg/kg)	2.97
	Mean value MB (LB–UB, mg/kg) ^b^	3.11 (3.11–3.12)
	Median value (LB–UB, mg/kg)	2.74
	P95 value, MB (LB–UB mg/kg)	7.70

^a^ Positive samples: 5-HMF level ≥ LOQ. ^b^ MB (LB–UB): Middle bound (lower bound–upper bound). LB: results below the LOQ were replaced with 0, MB: results below the LOQ were replaced with LOQ/2, UB: results below the LOQ were replaced with the value of LOQ.

**Table 3:** Distribution and concentrations of PAT in reconstituted pomegranate juice samples.

Food Products	Parameters	PAT
Pomegranate juice (*n* = 154)	Positive samples ^a^ *n* (%)	57 (37.0)
	Range (min–max, μg kg^−1^)	3.61–50.69
	Mean of positive samples (μg/kg)	14.48
	Mean value MB (LB–UB, μg/kg) ^b^	6.46 (5.36–7.56)
	Median value (LB–UB, μg/kg)	1.75 (0–3.50)
	P95 value, MB (LB–UB μg/kg)	23.93
	>ML ^c^, *n* (%)	1 (0.65)

^a^ Positive samples: PAT level ≥ LOQ. ^b^ MB (LB–UB): Middle bound (lower bound–upper bound). LB: results below the LOQ were replaced with 0, MB: results below the LOQ were replaced with LOQ/2, UB: results below the LOQ were replaced with the value of LOQ. ^c^ ML: Maximum level (50 μg kg^−1^).

**Table 4:** Estimated mean and 95th percentile (P95) dietary exposure to 5-HMF and PAT from pomegranate juice consumption among adults and children.

	Dietary Exposure (µg/kg bw/day)							
	Scenario 1				Scenario 2			
Analyte	Adults		Children		Adults		Children	
	Mean, MB (LB-UB)	P95, MB (LB-UB)	Mean, MB (LB-UB)	P95, MB (LB-UB)	Mean, MB (LB-UB)	P95, MB (LB-UB)	Mean, MB (LB-UB)	P95, MB (LB-UB)
5-HMF	2.358 (2.352–2.362)	5.828	8.530 (8.510–8.546)	21.08	0.375 (0.374–0.376)	0.927	1.141 (1.139–1.144)	2.821
PAT	0.005 (0.004–0.006)	0.018	0.018 (0.015–0.021)	0.066	0.001 (0.001–0.001)	0.003	0.002 (0.002–0.003)	0.009

MB (LB–UB): Middle Bound (Lower Bound–Upper Bound). LB, MB, and UB represent the assumptions where concentrations below the LOQ are replaced with 0, LOQ/2, and LOQ, respectively. When the LB and UB values are coincident, the range is not reported.

## Data Availability

The original contributions presented in this study are included in the article. Further inquiries can be directed to the corresponding author.
